# Impact of peripheral optical errors in age-related macular degeneration

**DOI:** 10.1016/j.optom.2026.100618

**Published:** 2026-06-11

**Authors:** Abinaya Priya Venkataraman, Robert Rosén, Marrie van der Mooren, Aixa Alarcón Heredia, Dmitry Romashchenko, Carmen Cánovas Vidal, Linda Lundström

**Affiliations:** a Department of Applied Physics, KTH (Royal Institute of Technology), Sweden; b Johnson & Johnson Vision, R&D, Groningen, Netherlands

**Keywords:** Peripheral vision, Resolution acuity, Contrast sensitivity, Off-axis aberrations, Phakic vs Pseudophakic optics, Age-related macular degeneration

## Abstract

**Purpose:**

Age-related macular degeneration (AMD) is a common ocular condition leading to reduced central visual function and increased dependency on the peripheral field of view for all visual tasks. This study aims to assess the effect of optical errors on peripheral vision in dry form AMD.

**Methods:**

High contrast resolution acuity and contrast sensitivity were measured in the 20° nasal visual field of fourteen subjects with advanced or early dry AMD. The measurements were performed through an adaptive optics vision simulator to simulate average phakic as well as pseudophakic peripheral optical errors.

**Results:**

Overall, inducing population average phakic optical errors resulted in better peripheral visual function than average pseudophakic optical errors for all measured subjects, both in high contrast resolution acuity and contrast sensitivity. Specifically, the mean differences between phakic and pseudophakic conditions were 0.16 logMAR in resolution acuity and 0.43 in logarithmic contrast sensitivity. These changes are larger than the results of studies on younger subjects with healthy vision.

**Conclusion:**

The study thereby concludes that reducing peripheral optical errors can improve peripheral vision, although further work is needed to determine how this translates to everyday visual performance. Interestingly, the improvement appears to be larger for AMD patients compared to healthy eyes.

## Introduction

Age-related macular degeneration (AMD) is the most common cause of vision loss in developed countries, especially for people over 50 years of age, and is estimated to affect 288 million people worldwide by 2040.^1^ AMD is characterized by dysfunctional central vision. Thereby, the peripheral field of view becomes important for many daily tasks where foveal vision is normally used, some people even develop new preferred retinal locations for, e.g., reading and face recognition. Sometimes magnification is used to alleviate the task at hand, but that comes at the cost of reduced visual field. In any case, the quality of the optical image on the peripheral retina is of particular importance for people with AMD irrespective of whether magnification is used or not.

It is well-known that the optical image quality in the eye decreases with eccentricity, mainly because of larger refractive errors and coma compared to the central visual field.^2^^,^^3^ Peripheral image quality also depends on the type and the design of any optical correction. Since cataract is a common co-existing ocular condition with AMD,^4^, ^5^, ^6^ many individuals with AMD have or will have intraocular lens implanted. Intraocular lenses may reduce the image quality on the peripheral retina compared to the natural phakic condition.^7^, ^8^, ^9^, ^10^, ^11^ Tabernero et al. found an increase in peripheral optical errors in eyes implanted with IOL from a variety of manufacturing companies and optical designs, both spherical and aspherical.^7^ The gradient index of the crystalline lens in the phakic eye is suggested to partly compensate for the peripheral astigmatism and the replacement of the crystalline lens with an IOL of constant index therefore results in higher peripheral astigmatism.^8^^,^^10^ Additionally, the peripheral average optical errors are shown to be more myopic with an increased astigmatism in pseudophakic eyes, leading to decreased peripheral image quality from 20° onward independently of IOL technology and eye configuration.^7^^,^^10^, ^11^, ^12^ Some studies have also investigated the visual effect of increased peripheral optical errors after IOL implantation in healthy patients.^11^^,^^13^ However, the visual impact of such increases in the peripheral optical errors have not been explored for patients with AMD, although they may be extra disadvantageous for these patients and for those who might develop AMD in the future.

Earlier studies have shown that peripheral detection acuity, low contrast resolution acuity, contrast sensitivity, and hazard detection can be improved by proper optical correction in people with normal macular function.^14^, ^15^, ^16^ These improvements can be even larger in persons with central visual field loss, who also show improvements in high contrast acuity.^17^, ^18^, ^19^, ^20^ However, those studies involved younger subjects who had suffered from less common forms of macular degeneration for most of their life. The purpose of the current study is therefore to evaluate the impact of simulated peripheral optical errors, typical of the phakic and the pseudophakic eye, on the peripheral visual function of people with the more common form of macular degeneration, AMD, debuting in the later part of life.

## Methods

Subjects with AMD (dry form) were recruited from a low vision clinic in Stockholm. The inclusion criteria were that the subject should have the dry form of AMD, no media opacities or any other ocular conditions, and be physically and mentally healthy enough to perform psychophysical measurements. Subjects with intraocular lenses were also included. As can be seen in Table 1, the recruited subjects were divided into two groups depending on the state of the AMD: nine subjects with advanced AMD severely affecting foveal vision, i.e. visual acuity of worse than 0.3 logMAR (decimal visual acuity <0.5), and five subjects with early AMD, i.e. close to normal foveal visual acuity (the relatively low acuity of subject eAMD1 is because of the lack of habitual correction after the cataract surgery). Average age was 79±7 years for the advanced AMD and 75±6 years for the early AMD. In total half of the subjects had a cataract surgery with monofocal intraocular lens implantation, whereas the other half were phakic. Informed consent was obtained beforehand, and the study protocol adhered to the tenets of the Declaration of Helsinki and was approved by the regional ethics committee.

**Table 1:** The 9 + 5 subjects with advanced and early state age-related macular degeneration (AMD and eAMD) participating in the study. The columns are: Age; Sex; Best eye = tested eye; Time since the diagnosis of AMD; Whether they have had cataract surgery or not; Whether they have developed any new preferred retinal location because of the central scotoma; Visual acuity (VA) with habitual refraction and the details of that refraction, if any.

**Subjects advanced AMD**	**Age [years]**	**Sex**	**Best eye**	**Diagnosis of AMD**	**Cataract surgery**	**Developed preferred retinal location**	**Decimal VA and habitual refraction in best eye**
AMD1	68	Male	Right	7–8 years ago	No	No	0.54
							(+2.75/−1.00×100)
AMD2	85	Female	Right	19 years ago	Yes	Yes several, best was 20° in inferior visual field	0.12
							(no, but in need)
AMD3	76	Female	Right	21 years ago	Yes	Yes, in inferior-nasal visual field (only 3–4° from scotoma)	0.22
							(no, but in need)
AMD4	83	Male	Right	20 years ago	No	Sometimes in inferior-temporal visual field	0.22
							(+2.50/−1.75×100)
AMD5	86	Female	Left	15–16 years ago	No	No	0.34
							(+0.50/−1.50×75)
AMD6	83	Female	Left	15 years ago	Yes	Sometimes in 6° inferior visual field	0.25
							(no)
AMD7	86	Female	Right	>25 years ago	Yes	Yes, close to 20° nasal visual field	0.14
							(+3.25/−1.50×133)
AMD8	76	Female	Right	2 years ago	Yes	Sometimes using the nasal visual field	0.09
							(no)
AMD9	67	Male	Right	3–4 years ago	No	Yes, in 7° nasal visual field	0.28
							(+3.50/−2.00×85)
**Subjects early AMD**	**Age [years]**	**Sex**	**Best eye**	**Diagnosis of AMD**	**Cataract surgery**	**Developed preferred retinal location**	**Decimal VA and habitual refraction in best eye**
eAMD1	81	Female	Right	10 years ago	Yes, surgery just a few months ago	-	0.63
							(no, but in need)
eAMD2	76	Male	Right	Just now	No	-	1.00
							(+3.75/−0.75×100)
eAMD3	81	Male	Right	1 year ago	No	-	0.8
							(+2.00/−0.50×70)
eAMD4	64	Male	Right	3 years ago	No	-	1.00
							(+0.75/0 x -)
eAMD5	72	Male	Right	1 year ago	Yes	-	1.00
							(no)

The peripheral visual function was evaluated monocularly in the 20° nasal visual field in the better eye of each subject. An adaptive optics vision simulator designed for peripheral vision evaluations was used to control the monochromatic optical errors of the peripheral viewing angle in a continuous closed loop.^21^ During the vision evaluation, the subject viewed a computer monitor via the deformable mirror of the system. The peripheral optical errors of the individual eye were compensated for, and two different optical conditions were induced with the adaptive optics vision simulator:

1) Simulated average phakic image quality representing the population average at 20° nasal visual field with −0.60 D in spherical equivalent, −0.50 D in J0 (i.e. a −1 D cylinder with axis 90°), and 0.09 µm horizontal coma for a 4 mm pupil.^2^
2) Simulated pseudophakic image quality at 20° nasal visual field found in an earlier study of implanted intraocular lenses^10^ and used in two other studies^9^^,^^16^ with −1.20 D in spherical equivalent, −1.45 D in J0 (i.e. a −2.9 D cylinder with axis 90°) and 0.09 µm horizontal coma for a 4 mm pupil.

High contrast grating resolution acuity cut-off was evaluated in all subjects for both optical conditions. Six of the 9 subjects with advanced AMD and 4 of the 5 subjects with early AMD also performed contrast sensitivity (CS) tests. The remaining four subjects did not perform the CS measurements because of fatigue.

The stimuli for these vision evaluations were high-contrast Gabor gratings oriented along the 45° or 135° meridian in accordance with Venkataraman et al.^22^ and presented on a calibrated monitor (cathode-ray tube with 10 bit greyscale). The spatial frequency and the contrast of the gratings were varied to determine the resolution acuity cut-off and the CS, respectively. The task of the subject was to identify the orientation of the presented grating in a two-alternative-forced-choice routine utilizing the Bayesian Ψ-method.^23^ Contrast sensitivity was evaluated at a spatial frequency logarithmically midway between 1 cpd (cycles per degree) and the acuity cut-off from the pseudophakic optical condition measurement. All measurements were repeated three times and the measurement time per subject was around 2.5 h (not including breaks given between the measurements).

## Results

**Fig. 1:**
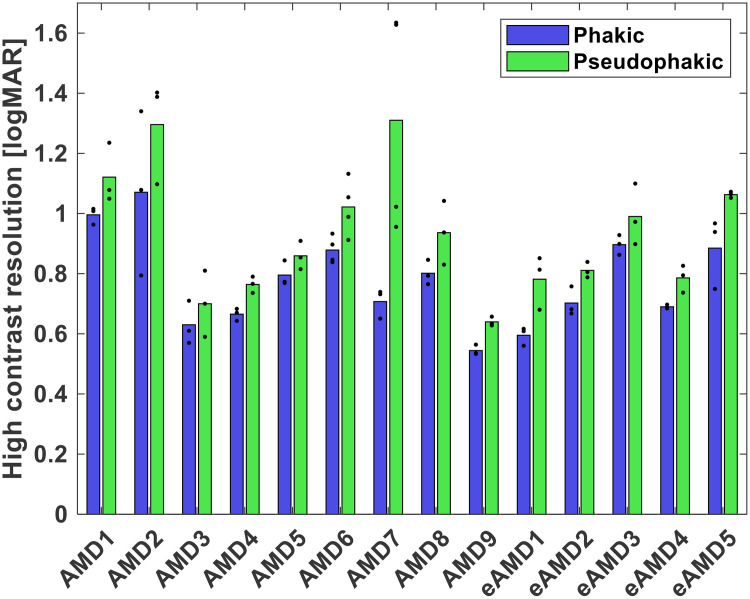
Peripheral resolution acuity cut-off (in logMAR) in the 20° nasal visual field for 9 subjects with advanced AMD (AMD1-AMD9) and 5 subjects with early AMD (eAMD1-eAMD5) under the two optical conditions: average phakic population errors at 20° nasal visual field (blue bars), and average pseudophakic errors at 20° nasal visual field (green bars). For each subject, the individual measurements are given as black dots and the bars denote the average.

**Fig. 2:**
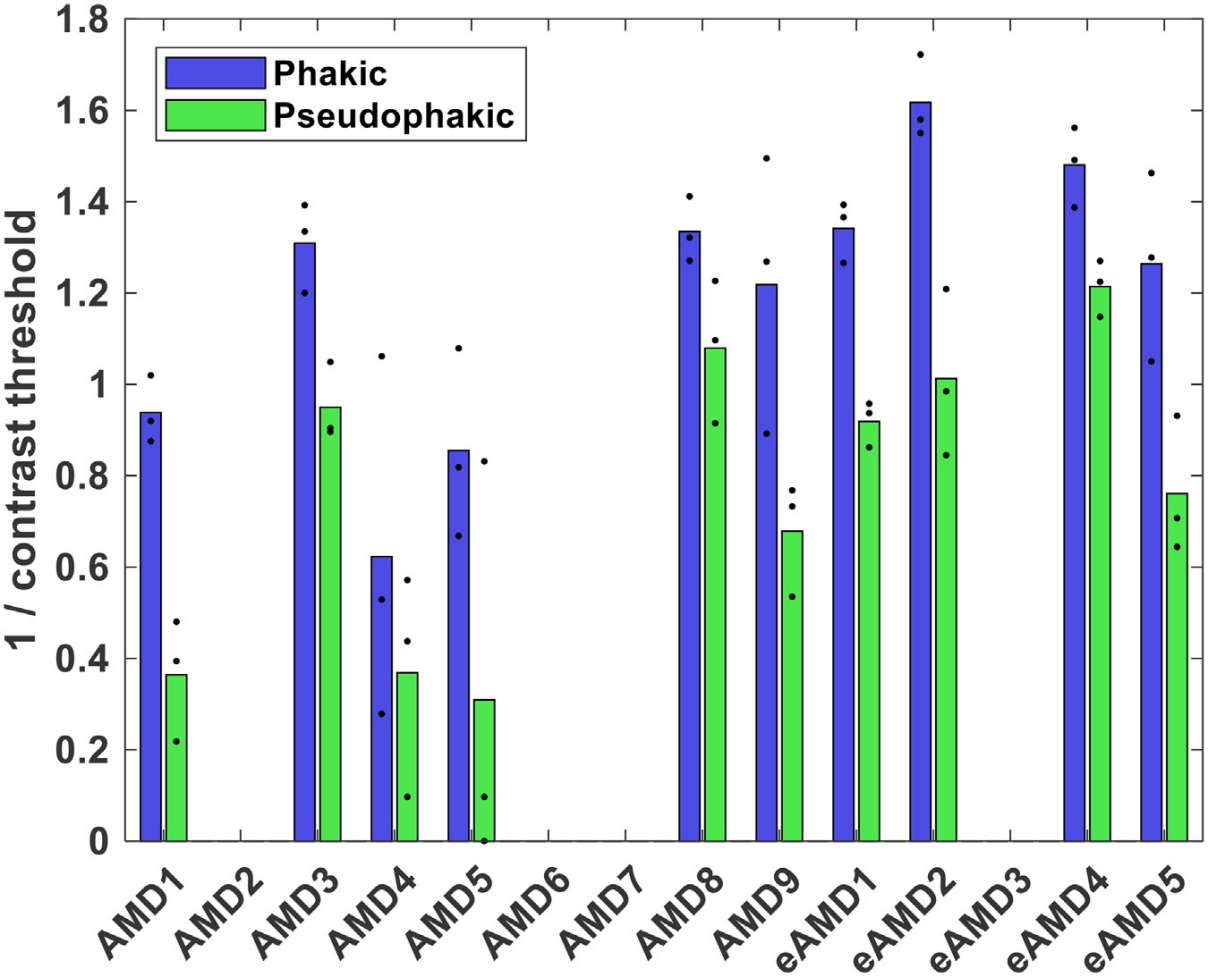
Peripheral logarithmic contrast sensitivity at 1.6 to 3.4 cycles per degree in the 20° nasal visual field for 9 subjects with advanced AMD (AMD1-AMD9) and 5 subjects with early AMD (eAMD1-eAMD5) under the two optical conditions: average phakic population errors at 20° nasal visual field (blue bars), and average pseudophakic errors at 20° nasal visual field (green bars). For each subject, the individual measurements are given as black dots and the bars denote the average. Contrast sensitivity for subject AMD2, AMD6, AMD7 and eAMD3 was not measured because of fatigue.

The resolution acuity cut-off and logarithmic contrast sensitivity (logCS) in the 20° nasal visual field for the simulated phakic and pseudophakic optical conditions for each subject are listed in Table 2 as average values and intervals of maximum and minimum measured value. The data are also visualized in Fig. 1, Fig. 2. Contrast sensitivity was not measured in subject AMD2, AMD6, AMD7 and eAMD3 due to fatigue after first performing the resolution acuity task (note that they were all above 80 years of age).

**Table 2:** Peripheral resolution acuity cut-off (in logMAR) and logarithmic contrast sensitivity (measured at the spatial frequency given in the last column in cycles per degree, cpd) in the 20° nasal visual field for 9 subjects with advanced AMD (AMD1-AMD9) and 5 subjects with early AMD (eAMD1-eAMD5) under two optical conditions: average simulated phakic population errors at 20° nasal visual field and average simulated pseudophakic errors at 20° nasal visual field. For each subject, the average results for the three repetitions are given, together with the minimum and the maximum measured values in parentheses. Contrast sensitivity for subject AMD2, AMD6, AMD7 and eAMD3 was not measured because of fatigue.

**Subjects**	**Peripheral high contrast resolution acuity cut-off in logMAR**		**Peripheral contrast sensitivity (logarithmic)**		
**Advanced AMD**	**Average phakic image quality**	**Average pseudophakic image quality**	**Average phakic image quality**	**Average pseudophakic image quality**	**Tested spatial frequency [cpd]**
AMD1	1.00	1.12	0.94	0.36	1.8
	(0.96 – 1.02)	(1.05 – 1.24)	(0.88 – 1.02)	(0.22 – 0.48)	
AMD2	1.07	1.30	-	-	-
	(0.79 – 1.34)	(1.10 – 1.40)			
AMD3	0.63	0.70	1.31	0.95	3.0
	(0.57 – 0.71)	(0.59 – 0.81)	(1.20 – 1.39)	(0.90 – 1.05)	
AMD4	0.67	0.76	0.62	0.37	3.0
	(0.64 – 0.68)	(0.74 – 0.79)	(0.28 – 1.06)	(0.10 – 0.57)	
AMD5	0.80	0.86	0.86	0.31	2.5
	(0.77 – 0.84)	(0.81 – 0.91)	(0.67 – 1.08)	(0 – 0.83)	
AMD6	0.88	1.02	-	-	-
	(0.84 – 0.93)	(0.91 – 1.13)			
AMD7	0.71	1.31	-	-	-
	(0.65 – 0.74)	(0.96 – 1.63)			
AMD8	0.80	0.94	1.33	1.08	2.0
	(0.77 – 0.85)	(0.83 – 1.04)	(1.27 – 1.41)	(0.91 – 1.23)	
AMD9	0.54	0.64	1.22	0.68	3.4
	(0.53 – 0.56)	(0.63 – 0.66)	(0.89 – 1.49)	(0.54 – 0.77)	

**Early AMD**	**Peripheral high contrast resolution acuity cut-off in logMAR**		**Peripheral contrast sensitivity (logarithmic)**		

eAMD1	0.60	0.78	1.34	0.92	2.23
	(0.56 – 0.62)	(0.68 – 0.85)	(1.27 – 1.39)	(0.86 – 0.96)	
eAMD2	0.70	0.81	1.62	1.01	2.16
	(0.67 – 0.76)	(0.79 – 0.84)	(1.55 – 1.72)	(0.84 – 1.21)	
eAMD3	0.90	0.99	-	-	-
	(0.86 – 0.93)	(0.90 – 1.10)			
eAMD4	0.69	0.79	1.48	1.21	2.22
	(0.68 – 0.70)	(0.74 – 0.83)	(1.39 – 1.56)	(1.15 – 1.27)	
eAMD5	0.88	1.06	1.26	0.76	1.6
	(0.75 – 0.97)	(1.05 – 1.07)	(1.05 – 1.46)	(0.64 – 0.93)

All 14 subjects showed improved peripheral visual function when the induced optical errors changed from typical pseudophakic to typical phakic levels. Compared to the simulated pseudophakic condition, the improvement in high contrast resolution cut-off was on average 0.16 logMAR (approximately 1.5 lines on Bailey-Lovie chart) for the whole group, which was found to be significant with p = 0.0008 in a paired sample *t*-test. Separately, the advanced AMD showed an improvement of 0.17 logMAR with five subjects showing partially overlapping data intervals, and the early AMD had 0.13 logMAR with one subject showing partially overlapping data intervals. For the 10 subjects who performed the CS measurements the average improvement from the pseudophakic to the phakic condition was 0.43 logCS (e.g. from CS 4 to 10 at 2–3 cpd), which was found to be significant with p = 0.000004 in a paired sample *t*-test. Separately, the six subjects with advanced AMD showed an improvement of 0.42 logCS with only subjects AMD4 and AMD5 having partially overlapping data intervals, and the four subjects with early state AMD had 0.45 logCS without any partially overlapping data intervals.

## Discussion

This study shows that the peripheral visual function in the 20° nasal visual field of 14 subjects with dry form AMD improves when reducing peripheral optical errors from simulated typical pseudophakic to typical phakic level, corresponding to a change in peripheral refraction from (+0.25 DS / −2.90 DC x 90) to (−0.10 DS / −1.00 DC x 90). These improvements in peripheral vision are in line with previous studies on the correction of peripheral refractive errors in the 20° nasal visual field.^15^, ^16^, ^17^, ^18^, ^19^, ^20^^,^^24^ In Table 3 the improvements found in the current study are compared with the results of similar studies on younger subjects with normal healthy vision, one study using the exact same measurement protocol and instrumentation.^16^ As can be seen, both the advanced and the early AMD subgroups showed larger average improvements with the change in peripheral refraction from pseudophakic to phakic levels than those reported for younger healthy subjects.

**Table 3:** Comparison of the average vision improvement when correcting from typical pseudophakic optical errors to phakic optical errors (2nd and 4th column) and further to full adaptive optics correction (3rd and 5th column) at 20° off-axis in different subject groups (data on healthy young eyes from other studies) .^16^^,^^25^

**Subjects**	**Improvement in peripheral high contrast resolution acuity cut-off in logMAR**		**Improvement in peripheral contrast sensitivity (logarithmic)**	
	**From pseudophakic to phakic**	**From phakic to full adaptive optics correction**	**From pseudophakic to phakic**	**From phakic to full adaptive optics correction**
Advanced AMD	0.17	0.03	0.42 @ 1.8 – 3.4 cpd	0.20 @ 1.8 – 3.4 cpd
Early AMD	0.13	-	0.45 @ 1.6 – 2.2 cpd	-
Healthy eyes	0.08 (data from ref 16)	0.03 (data from ref 25)	0.25 @ 1 cpd (data from ref 16)	-

To further investigate possible visual benefits with improved peripheral optical quality, Table 3 also includes data on the 9 advanced AMD subjects when measured with full adaptive optics correction, i.e. when both lower and higher order monochromatic aberrations were corrected. On individual level, 7 out of the 9 advanced AMD subjects showed slightly better peripheral resolution acuity with full correction, similarly to healthy eyes. However, for contrast sensitivity, all of the six tested subjects improved with full optical correction, with an average of 0.20 in logCS. This means that, for at least some people with AMD, contrast sensitivity benefits with full optical correction can be of similar magnitude as the improvement when changing from the pseudophakic to the phakic optical condition in healthy eyes – further suggesting that reduction of peripheral optical errors is more important in AMD compared to healthy young eyes.

The results of this study imply that, although the population average of naturally occurring phakic peripheral defocus, astigmatism, and coma in the 20° nasal visual field does not hamper peripheral high-contrast resolution cut-off, larger optical errors may. Optical correction for peripheral vision can thereby be particularly relevant for people with larger than average phakic optical errors, as for example in the case of pseudophakia. The fact that 12 of the 14 AMD subjects show improvements of about 0.1 logMAR or more in resolution with the phakic compared to the pseudophakic condition is in good agreement with the threshold of −1.50 D astigmatism for 0.1 logMAR reduction suggested by Lewis et al.^24^ Contrast sensitivity at low spatial frequencies for all 10 tested AMD subjects also benefitted from optical correction and showed larger improvements than young subjects with healthy vision.

Translating improvements in peripheral visual acuity and contrast sensitivity to functional impact is not straightforward.^11^ However, in our previous study on healthy eyes, using the same visual testing protocol and optical conditions, the induced pseudophakic condition impaired hazard detection during a driving-movie at 20° eccentricity (i.e. more false positives and misses, and longer reaction time).^16^ Such functional testing was not included in the present study, as tasks of this nature are generally less representative for the AMD population. Therefore, given the larger visual effects found in the present study, future work is needed to understand how these visual improvements could translate into functional benefits for AMD patients. In this context, it should also be mentioned that a new IOL design was recently proposed to address peripheral optics after cataract surgery.^26^ While in healthy eyes this lens has demonstrated a reduction of peripheral errors as compared to current IOL technology, improvements of peripheral contrast sensitivity have been shown only at 40°,^27^ without demonstrating improvements in the overall driving performance.^28^ Additional studies are also needed to assess the potential functional effect of this IOL design for AMD patients.

The major limitations of this study are the low number of subjects and the variation in ocular status, which is quite common in these types of studies with lengthy psychophysical measurements on elderly. This means that individual outliers and variations may have a larger effect on the average results. Four-way analysis of variance tests (optical condition / individual / cataract surgery / AMD severeness) were therefore performed to assess whether there were any systematic differences between subject groups – but no significant differences were found neither between the advanced and early AMD groups nor between subjects who had and had not undergone cataract surgery. Furthermore, it should be noted that the test sequence was only randomized with respect to the optical condition (phakic/pseudophakic), and high contrast resolution acuity was tested before contrast sensitivity for all subjects. Additionally, we want to highlight that one and the same peripheral retinal location and the same simulated optical errors were used for all subjects to enable direct comparison to studies on other subject groups. This standardized protocol also reduced between-subject variability associated with eccentricity-dependent differences in retinal location and individual peripheral optical errors, which was particularly important in our relatively small cohort. However, an individualized approach based on each subject’s preferred retinal location and own peripheral optics would provide a better estimate of patient-specific benefit and should be evaluated in future studies.

## Conclusions

Reducing peripheral refractive errors from typical pseudophakic to typical phakic level significantly improves high contrast resolution acuity and contrast sensitivity in the 20° nasal visual field of patients with AMD. These improvements surpass those reported in studies involving younger subjects with normal vision. Therefore, the findings suggest that reducing peripheral optical errors can improve peripheral vision. Improvements appear to be larger for AMD patients compared to healthy eyes, although their magnitude may vary between individuals and across different visual tasks.

## Declarations of competing interest

Contract research for Johnson & Johnson Vision: APV, LL

Employment at Johnson & Johnson Vision: RR, MM, AA, DR, CC
